# SCO6992, a Protein with β-Glucuronidase Activity, Complements a Mutation at the *abs*R Locus and Promotes Antibiotic Biosynthesis in *Streptomyces coelicolor*

**DOI:** 10.4014/jmb.2108.08001

**Published:** 2021-09-25

**Authors:** Xue-Mei Jin, Mu-Yong Choi, Maral Tsevelkhoroloo, Uhnmee Park, Joo-Won Suh, Soon-Kwang Hong

**Affiliations:** 1 Department of Bioscience and Bioinformatics, Myongji University, Yongin 17058, Republic of Korea; 2 Characteristic Industry Development Center of Yanbian, Jilin Province, P.R. China; 3 Department of Biotechnology. The University of Suwon, Suwon 18323, Republic of Korea

**Keywords:** *Streptomyces coelicolor*, SCO6992, actinorhodin, undecylprodigiosin, β-glucuronidase

## Abstract

*Streptomyces coelicolor* is a filamentous soil bacterium producing several kinds of antibiotics. *S. coelicolor* abs8752 is an *abs* (antibiotic synthesis deficient)-type mutation at the *abs*R locus; it is characterized by an incapacity to produce any of the four antibiotics synthesized by its parental strain J1501. A chromosomal DNA fragment from *S. coelicolor* J1501, capable of complementing the abs- phenotype of the abs8752 mutant, was cloned and analyzed. DNA sequencing revealed that two complete ORFs (SCO6992 and SCO6993) were present in opposite directions in the clone. Introduction of *SCO6992* in the mutant strain resulted in a remarkable increase in the production of two pigmented antibiotics, actinorhodin and undecylprodigiosin, in *S. coelicolor* J1501 and abs8752. However, introduction of *SCO6993* did not show any significant difference compared to the control, suggesting that SCO6992 is primarily involved in stimulating the biosynthesis of antibiotics in *S. coelicolor*. *In silico* analysis of SCO6992 (359 aa, 39.5 kDa) revealed that sequences homologous to SCO6992 were all annotated as hypothetical proteins. Although a metalloprotease domain with a conserved metal-binding motif was found in SCO6992, the recombinant rSCO6992 did not show any protease activity. Instead, it showed very strong β-glucuronidase activity in an API ZYM assay and toward two artificial substrates, *p*-nitrophenyl-β-D-glucuronide and AS-BI-β-D-glucuronide. The binding between rSCO6992 and Zn^2+^ was confirmed by circular dichroism spectroscopy. We report for the first time that SCO6992 is a novel protein with β-glucuronidase activity, that has a distinct primary structure and physiological role from those of previously reported β-glucuronidases.

## Introduction

*Streptomycetes* is a group of gram-positive and filamentous soil bacteria with a unique and complex differentiation process that includes simultaneous morphological (sporulation) and physiological (secondary metabolism) differentiation. Many scientific findings have elucidated that the two differentiation processes are genetically closely related and thus controlled concurrently by many regulatory factors. Generally, genes for secondary metabolite formation and morphological differentiation are clustered in the chromosome, and their expression is precisely controlled by a pathway-specific regulatory gene(s) within the cluster as well as by global regulatory genes [1-3].

Several classes of mutants that simultaneously block the synthesis of more than one antibiotic constitute a good genetic evidence of a global regulatory system. *Streptomyces coelicolor* has been known to produce four genetically and structurally distinct antibiotics: the pigmented antibiotics actinorhodin and undecylprodigiosin and the unpigmented antibiotics methylenomycin and CDA (Ca^2+^-dependent antibiotic) [4]. According to an *S. coelicolor* genome sequence analysis, 18 gene clusters are expected to code exclusively for enzymes characteristic of its secondary metabolism [5]. *abs* (antibiotic synthesis deficient) mutants, for example, affect global regulatory genes involved only in physiological differentiation. Among them, *abs*^-^ mutations *abs*A^-^, *absB*^-^, and *absC*^-^ were found to lack all four antibiotics, but retained normal sporulation. The molecular nature of the *absA1/A2* gene products that can restore the bacterial ability for biosynthesizing all four antibiotics was identified as a two-component regulatory system, composed by the sensor histidine kinase AbsA1 and the aspartate kinase response regulator AbsA2 [4]. The phosphorylated AbsA2 is known to act as a global negative regulator of antibiotic biosynthesis in *S. coelicolor* [6]. Besides, the global defect in antibiotic synthesis in the *absB*^-^ mutant was due to a deficiency in RNase III (*rnc*) [7] which regulates the production of antibiotics through its double strand-specific endoRNase activity [8]. Finally, the *absC* locus exerts pleiotropic, zinc-dependent effects on antibiotic biosynthesis [9]. Interestingly, it was shown that the production of actinorhodin and undecylprodigiosin was abolished when *absC* was deleted, but this phenomenon was only observed when zinc concentrations were low. AbsC also represses the expression of the coelibactin biosynthetic gene cluster by binding to an operator sequence adjacent to the zinc-responsive regulatory protein Zur binding site [9]. Although these three mutations such as *abs*A^-^, *absB*^-^, and *absC*^-^ are categorized into the same *abs*^-^ mutation, the molecular properties of them are quite different, implying that the control machinery for secondary metabolism in *Streptomyces* is extraordinarily complex [10].

We previously reported on the genetic analysis of a new *abs*-like mutation at the *abs*R locus of the mutant strain *S. coelicolor* abs8752 [11]. The *abs*R^-^ mutation was complemented with multiple copies of the *actII-ORF4* gene encoding for a pathway specific regulator for actinorhodin biosynthesis [3] and by the *afsR* gene encoding a global regulator for morphological and physiological differentiation [12], but not with *abs*A. Therefore, as *abs*R was considered an *abs*-type mutation distinct from *abs*A^-^ or *absB*^-^ mutations [11], the chromosomal DNA fragment that could complement strain abs8752 for antibiotic biosynthesis was cloned from its parental strain, *S. coelicolor* J1501. In this fragment, two complete ORFs, namely ORF-1 (equivalent to SCO6992) and ORF-2 (equivalent to SCO6993), were identified. In this report, we describe the functional analysis of the *SCO6992* gene; a gene that is able to restore and stimulate antibiotic biosynthesis in *S. coelicolor*.

## Materials and Methods 

### Bacterial Strains and Plasmids

*S. coelicolor* J1501 (*hisA1*^-^ *uraA1*^-^ *strA1* SCP1^-^ SCP2^-^ Pgl^-^) derived from *S. coelicolor* A3(2) and *Streptomyces lividans* TK24 were obtained from the John Inns Institute, U.K. [13]. *S. coelicolor* abs8752 (an *abs*R^-^ mutant that was selected as a mutant blocked in antibiotic biosynthesis after treating J1501 with *N*-methyl-*N*′-nitro-*N*-nitrosoguanidine) was kindly given by Dr. Wendy Champness from Michigan State University [14]. The *Escherichia coli*-*Streptomyces* shuttle vector pWHM3 [15] was used for cloning and expression in *Streptomyces* spp.. *E. coli* JM109 was used for genetic manipulations.

### Media and Culture Conditions

*E. coli* was maintained on M9 minimal agar and routinely cultured in Luria broth [13] at 37°C with agitation. *Streptomyces* strains were maintained on R2YE agar or liquid medium at 30°C [13]. For the cultivation of *S. coelicolor* strains, histidine and uracil were used as supplements at final concentrations of 50 and 7.5 μg/ml, respectively [11].

### Enzymes and Chemicals

Restriction endonucleases and other DNA modifying enzymes were purchased from DyneBio Inc. (Korea). Unless specified, any other fine chemical was from Sigma-Aldrich Corporation (USA).

### DNA Manipulation and Transformation

DNA preparation and manipulation were performed by the methods described by Kieser *et al*. [13]. All kits and enzymes were used according to the manufacturer's recommendations. Preparation of competent *E. coli* cells and transformations were performed as described [13]. Protoplast preparation and PEG-mediated transformation of *Streptomyces* cells were carried out as described [13]. *Streptomyces* transformants were selected by overlaying with 0.6% soft agar containing 25 μg/ml of thiostrepton.

### Cloning of a DNA Fragment with absR-Complementing Activity

**Fig. 1:**
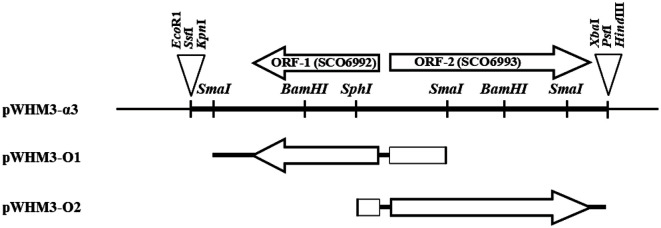
Schematic representation of the genes within the insert in pWHM3-α3 and fragments used in the construction of expression vectors for each open reading frame (ORF). Arrows indicate individual ORFs, and arrowheads mark the corresponding stop codons. DNA fragments derived from *S. coelicolor* J1501 are indicated by thick lines, and vector fragments, including the multiple cloning site, are depicted by thin lines. For the construction of pWHM3-O1 and pWHM3-O2, DNA fragments digested with *Sma*I-*Sma*I or *Sph*I–*Pst*I, respectively, were subcloned into pWHM3, an *E. coli*-*Streptomyces* shuttle vector.

*S. coelicolor* J1501 was cultured in R2YE medium and used for chromosomal DNA isolation, as described previously [16]. To clone the *abs*R^-^ complementing DNA fragment, the chromosomal DNA was partially digested with restriction enzyme *Sau*3A1, and separated elctrophoretically on a 1% agarose gel. The pool of 4-6 kb DNA fragments recovered from the gel was ligated with the pWHM3 vector digested with *Bam*HI to construct a mini library by shotgun cloning. Ligated plasmids were transformed into *S. coelicolor* abs8752. Among the 1,650 transformants, one colony with an *abs*^+^ phenotype (pigment production) was selected to isolate the recombinant plasmid, pWHM3-α3, which carried a 3,919-bp insert (GenBank accession number; AF136167) that included two ORFs, according to DNA sequencing analysis (Fig. 1). The two ORFs, ORF-1 and ORF-2, were identified as SCO6992 and SCO6993 annotated from the *S. coelicolor* A3(2) genome sequence [5], respectively.

### Quantitative Analysis of Antibiotic Production

Each ORF was individually subcloned. A *Sma*I fragment, 1,894 bp long, containing the entire SCO6992 including its promoter region was subcloned into pWHM3 to yield pWHM3-O1. In addition, a *Sph*I-*Pst*I digested fragment, 2,711 bp long, containing the entire SCO6993 and its promoter region was cloned into pWHM3 to yield pWHM3-O2 (Fig. 1). The capacity of producing pigmented antibiotics actinorhodin (blue) and undecylprodigiosin (red) by *S. coelicolor* and *S. lividans* TK24 transformants could be easily checked with the naked eye on a solid medium. Quantitation of actinorhodin and undecylprodigiosin in liquid culture was performed as previously described [12]. Briefly, an exponential phase culture of *S. coelicolor* was transferred to 100 ml of R2YE medium containing 25 μg/ml of thiostrepton in a 500 ml baffled flask, and incubated at 30°C on a reciprocal shaker. At indicated intervals, 5 ml aliquots of the culture broth were extracted with 5 ml of chloroform for 30 min at 20°C, with shaking. Then, 5 ml of 1 N NaOH was added, and the tubes were vortexed and spun in a microcentrifuge for 15 s. Actinorhodin, distributed to the aqueous phase, had a blue color at alkaline pH, and was quantified by measuring the absorbance at 640 nm (A_640_). Undecylprodigiosin had a yellow color at alkaline pH and remained in the chloroform layer; it was acidified with HCl, and turned to red at acidic pH. It was quantified by measuring the A_530_. The concentrations of actinorhodin and undecylprodigiosin were calculated using the molar extinction coefficients ε_640_ = 25,320 M^-1^ cm^-1^ and ε_530_ = 100,150 M^-1^ cm^-1^, respectively.

### Expression and Purification of the MBP-SCO6992 Fusion Protein in *E. coli*

The pMAL-c2x vector was used to express the SCO6992 protein in *E. coli*. The *SCO6992* DNA fragment (1,080 bp long) was amplified by PCR as previously described [17] using primers SCO6992-forward (5'-GTCGACATGACCGCACGCTACTGCTCG-3', the *Sal*I site is underlined) and SCO6992-reverse (5'-AAGCTTTCACCAGTACATGACCGCCGTC-3', the *Hind*III site is underlined). The amplified fragment was then cloned into pMAL-c2x digested with *Sal*I/*Hind*III restriction enzymes. The resulting plasmid, pMAL-c2x-SCO6992, was designed to express SCO6992 fused to MalE maltose binding protein (MBP). *E. coli* JM109/pMAL-c2x-SCO6992 cells were inoculated in 1 L of rich medium (Luria broth containing 0.2% glucose) with ampicillin (50 μg/ml) and cultured at 37°C with shaking. When the optical density (OD_600_) of the culture broth was 0.4, isopropyl-D-thiogalactopyranoside (0.3 mM) was added to induce SCO6992 expression. After culturing for 3 h, the cells were harvested by centrifugation (7,000 ×*g*, 10 min, 4°C) and resuspended in buffer A (20 mM Tris-HCl pH 7.4, 200 mM NaCl, 1 mM EDTA, 10 mM β-mercaptoethanol). Cells were then disrupted by sonication and centrifuged at 15,000 ×*g* for 30 min at 4°C to prepare a cell-free lysate that was used for the purification of the MBP-SCO6992 fusion protein (MBP-SCO6992) by using an MBP.Bind Agarose Resin (ELPIS Biotech, Korea). The purified MBP-SCO6992 protein, eluted with buffer A containing 10 mM maltose, was concentrated using an Amicon ultra centrifugal filter 50K device, followed by a buffer exchange for 10 mM Tris-HCl (pH 7.9) for further experimentation. The purified protein was analyzed by 0.1% sodium dodecyl sulfate-10% polyacrylamide gel electrophoresis (SDS-PAGE).

### Circular Dichroism (CD) Spectroscopy Analysis

To investigate the complexes formed between the MBP-SCO6992 protein and different metal ions, CD spectroscopy analysis was performed using a J-815 spectropolarimeter (Jasco Corp., Japan), using a wavelength range of 200-260 nm, and a quartz cell with a path length of 0.1 mm [18]. Scanning was performed 10 times per sample at a scan rate of 100 nm per min. Measurements were done at 20°C, with 1 μg/μl protein and 2 mM metal ion (either Fe^2+^, Zn^2+^, Mn^2+^, Ca^2+^, Mg^2+^, or Cu^2+^).

### Protease Activity Determination

A skim milk hydrolysis assay was performed to detect protease activity. Briefly, 20 μl of purified MBP-SCO6992 was dropped onto a 1% skim milk agar plate, and the formation of a clear zone was observed after incubation at 37°C for 24 h.

The azocasein hydrolysis method was then used to measure the total protease activity of the purified MBP-SCO6992 as follows: 120 μl of protein was mixed with 480 μl of 1% azocasein solution in 25 mM Tris-HCl buffer (pH 8.0), and left to react at 37°C for 30 min. Then, the reaction was terminated by adding 600 μl of a 10% (w/v) trichloroacetic acid solution. After centrifugation at 15,000 ×*g* for 10 min, 800 μl of the supernatant was taken and mixed with 200 μl of 1.8 N NaOH. The concentration of azo dye, a hydrolysis product, was measured spectrophotometrically at 420 nm (A_420_). The effect of metal ions on protease activity were also investigated by adding various metal ions to the reaction mixture.

### Enzyme Activity Analysis

An API ZYM kit (bioMérieux, France) was used to investigate the enzymatic activity of the purified protein. Following the manufacturer’s instructions, 200 μl of purified protein was added to each plate well together with the corresponding reaction reagents. The presence or absence of enzymatic activity was determined according to the resultant color change.

### Measurement of β-Glucuronidase Activity

To measure the β-glucuronidase activity of the purified protein, an artificial substrate *p*-nitrophenyl-β-D-glucuronide was used [19]. The reaction mixture (600 μl) contained 83 mM acetate buffer (pH 4.5), 1.67 mM *p*-nitrophenyl-β-D-glucuronide, and 200 μl of purified protein (after the final concentration step). After incubating the reaction mix at 37°C for 1 h, 400 μl of 2.5 M 2-amino-2-methyl-1,3-propanediol was added to stop the reaction, and the concentration of p-nitrophenol, a hydrolysis product, was measured at 415 nm (A_415_).

For β-glucuronidase zymographic analysis, protein samples were loaded onto a 9% native polyacrylamide gel. Electrophoresis was performed in 25 mM Tris-HCl (pH 8.1) buffer containing 192 mM glycine, at 4°C and 160 V for 2 h. After washing twice with triple distilled water, the gel was immersed in 100 mL of 0.2 M sodium acetate buffer (pH 5.2) containing 40 mg of naphthol AS-BI-β-D-glucuronide (an artificial substrate) and 40 mg of Fast Garnet, and the color change on the zymogram was observed [20].

## Results

### Complementation of the *abs*R^-^ Mutation by the pWHM3-α3 Clone

*S. coelicolor* abs8752 was used as the host strain to identify the gene complementing the *abs*^-^ phenotype. First, one thiostrepton-resistant transformant with *abs*^+^ phenotype on an agar plate was selected, and the recombinant plasmid (named pWHM3-α3) carrying a 4-kb insert was isolated from it (Fig. 1).

**Fig. 2:**
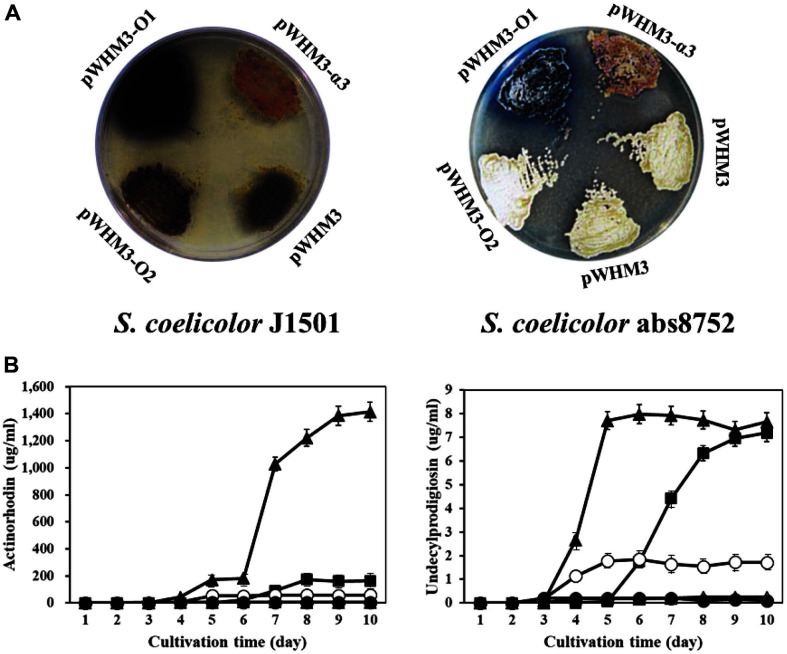
Effect of cloned DNA fragments on pigmented antibiotic production in *Streptomyces coelicolor*. (**A**) Production of pigmented antibiotics by *S. coelicolor* J1501 (left) and *S. coelicolor* abs8752 (right) transformants carrying different plasmids. Transformants were grown on R2YE plates for 5 days before being photographed. Compared to wild type *S. coelicolor* J1501/pWHM3, *S. coelicolor* *abs*8752/pWHM3 did not produce any pigment. However, *S. coelicolor* *abs*8752/ pWHM3-α3 shows a dark-reddish color, indicating a restoration in the production of pigmented antibiotics. Complementation with individual ORFs showed that only ORF-1 (plasmid pWHM3-O1) could remarkably stimulate antibiotic production (especially actinorhodin pigment) in both strains. (**B**) Pigmented antibiotic production by *S. coelicolor* cultured in R2YE broth. Culture samples were taken at regular intervals of time and processed, as described in Materials and Methods. Then, quantification of actinorhodin (left) and undecylprodigiosin (right) was done by measuring the absorbance at 640 and 530 nm, respectively. ○, *S. coelicolor* J1501/pWHM3; ●, *S. coelicolor* abs8752/pWHM3; ■, *S. coelicolor* abs8752/ pWHM3-α3; ▲, *S. coelicolor* abs8752/pWHM3-O1; △, *S. coelicolor* abs8752/pWHM3-O2. All experiments were repeated at least 3 times, and their average values were calculated.

pWHM3-α3 was able to restore actinorhodin and undecylprodigiosin biosynthesis in *S. coelicolor* abs8752 on R2YE plates (Fig. 2A). Judging by the color of the bacterial growth, undecylprodigiosin (red color) and actinorhodin (blue color) were mixed, though the final color was more similar to that of undecylprodigiosin than to that of actinorhodin. The transformant also showed that its ability to produce the non-pigmented antibiotics methylenomycin and CDA was restored (data not shown). This result strongly supports that the cloned fragment has the ability to complement the pleiotropic antibiotic production deficiency of *S. coelicolor* abs8752.

### Molecular Analysis of the pWHM3-α3 Clone

Nucleotide sequencing revealed that two complete ORFs (ORF-1 and ORF-2), transcribed in opposite directions, were included in the 3,919-bp insert (GenBank accession number: AF136167) of pWHM3-α3. The genes encoding ORF-1 (protein_id: AAF19103.1, 359 amino acids, 39.5 kDa) and ORF-2 (protein_id: AAF19104.1, 606 amino acids, 65.7 kDa) were registered in GenBank as *abs*R1 and *abs*R2, respectively, in the sense that they complemented the *abs*R mutation.

### Chromosomal Location of the ORF-1 and ORF-2 in *S. coelicolor*

We previously reported that the *abs*R locus exists near *abs*A locus on the chromosomal DNA of *S. coelicolor*. Based on the genomic sequence of *S. coelcicolor* A3(2), AbsA1 (SCO_3_225) and AbsA2 (SCO_3_226) were located between the base sequences 3536945 and 3539347, and ORF-1 (SCO6992) and ORF-2 (SCO6993) were placed between 7759825 and 7762803 of chromosomal DNA. Therefore, it was assumed that *abs*A1/A2 and putative *abs*R1/R2 are separated by more than 4.2 million bp on the chromosome. In addition, the nucleotide sequencing of the DNA fragments revealed that *S. coelicolor* J1501 and abs8752 strains had the same nucleotide sequence in ORF-1 and ORF-2 coding region. These results clearly indicate that although ORF-1 and ORF-2 can restore the antibiotic-producing ability of *abs*R mutant, they are different from the actual gene corresponding to the *abs*R locus. Therefore, we marked SCO6992 and SCO6993 assigned by genomic sequencing for ORF-1 and ORF-2, respectively.

### Gene Dosage Effect of SCO6992 and SCO6993 on Antibiotics Production

The introduction of pWHM3-O1 containing *SCO6992* in *S. coelicolor* abs8752 and *S. coelicolor* J1501 could restore and remarkably stimulate pigment production (mainly blue) on R2YE plates, respectively; pigment production was not observed among transformants carrying either pWHM3-O2 (containing *SCO6993*) or pWHM3 (Fig. 2A).

When the amount of actinorhodin and undecylprodigiosin produced in R2YE liquid media at day 10 was compared, it was seen that *S. coelicolor* abs8752/pWHM3-O1 produced 24.2 times more actinorhodin than transformants with either pWHM3 or pWHM3-O2, and 8.6 times more actinorhodin than that produced by *S. coelicolor* abs8752/pWHM3-α3 (Fig. 2B, left). Moreover, the amount of undecylprodigiosin produced by *S. coelicolor* abs8752/pWHM3-O1 was slightly higher than that produced by *S. coelicolor* abs8752/pWHM3-α3, and at least 4 times higher than those of the transformants with either pWHM3 or pWHM3-O2 (Fig. 2B, right). This fact clearly indicates that the *SCO6992* gene is responsible for inducing the biosynthesis of actinorhodin and undecylprodigiosin in *S. coelicolor* strains. Interestingly, the stimulatory effect by *SCO6992* on antibiotic production (especially on actinorhodin) was severely suppressed by the presence of *SCO6992* as shown in *S. coelicolor* abs8752 and *S. coelicolor* J1501 transformed with pWHM3-α3, implying that SCO6993 may be a repressor.

### *In silico* Analysis of SCO6992

The top 100 genes with high homology to SCO6992, resulting from a Blastp search (BLAST: Basic Local Alignment Search Tool (nih.gov)), belonged to *Streptomyces* species; their identity ranged between 77% and 99%. However, they are all annotated as hypothetical proteins and their functions have not been elucidated. For example, SCO6992 has 99% identity with the unidentified AIJ11880.1 protein (361 aa) from *S. lividans* TK24. Moreover, many hypothetical proteins from various gram-positive and gram-negative bacteria and even fungi were also listed as homologous to SCO6992 with lower scores, suggesting that SCO6992 orthologue is widely distributed in bacteria and other organisms.

**Fig. 3:**
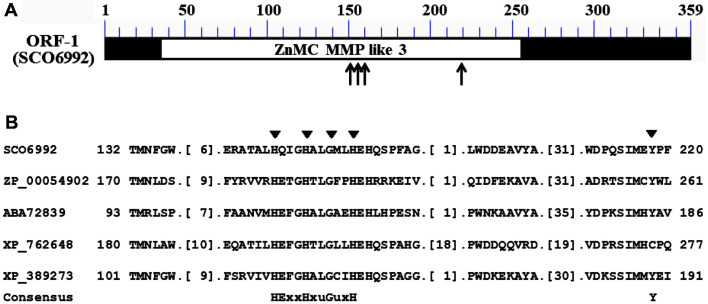
Conserved metalloprotease domain in SCO6992 and sequence alignment for identifying its metal binding motif. (**A**) Schematic representation of the zinc-dependent metalloprotease (MMP-like sub-family 3) domain found in SCO6992. The conserved region is represented by an open box, whereas the most highly conserved region, bearing three His and one Tyr residues, is indicated by arrows. (**B**) Alignment of several amino acid sequences to identify the putative metal binding motif in SCO6992. The metal binding domain cd04327, with the conserved sequence HExxHxuGuxH (u = bulky hydrophobic), has been reported in various zinc-dependent metalloproteases. The most conserved residues, *i.e.*, three His, one Gly, and one Tyr residues, are indicated with black triangles. Aligned proteins belong to *Magnetospirillum magnetotacticum* MS-1 (ZP_00054902), *Pseudomonas fluorescens* Pf0-1 (ABA72839), *Ustilago maydis* 521 (XP_762648), and *Fusarium graminearum* PH-1 (XP_389273).

Interestingly, SCO6992 has an MMP-like sub-family 3 domain (cd04327), common in zinc-dependent metalloproteases, in the region spanning Ala-33 through Pro-254, with an E-value of 2.36 × e^-88^ [21]. The MMP-like sub-family 3 domain is constituted by a group of bacterial and fungal metalloprotease domains whose structure is similar to those of matrix metalloproteases and astacin (Fig. 3A). Additionally, SCO6992 has a well-conserved metal binding domain composed of three His-residues that are important elements of the active site of metalloproteases (Fig. 3B) [22].

### Expression and Purification of an MBP-SCO6992 Fusion Protein

**Fig. 4:**
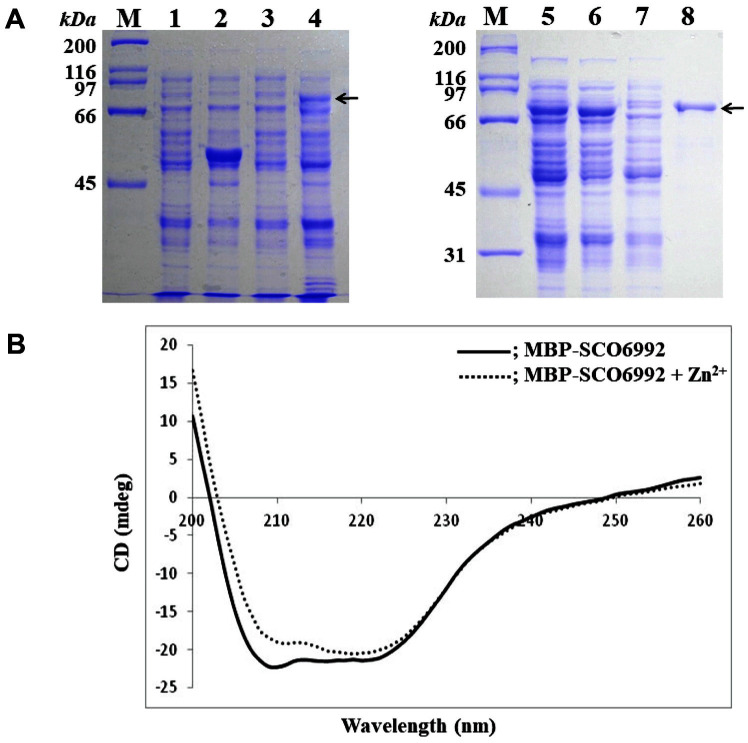
Purification and confirmation of metal-binding activity of the MBP-SCO6992 recombinant protein. (**A**) Sodium dodecyl sulfate-polyacrylamide gel electrophoresis of proteins during purification by affinity chromatography. MBP-SCO6992 was overexpressed in *E. coli* JM109/pMAL-c2x-SCO6992 after IPTG induction (lanes 1–4, left). Then, the recombinant protein was purified from the cell-free extract by an amylose-affinity column chromatography (lanes 5–8, right). The MBP-SCO6992 protein is indicated by arrows. Lanes: M, molecular weight size markers; 1, *E. coli* JM109/pMAL-c2x before induction; 2, *E. coli* JM109/pMAL-c2x after induction; 3, *E. coli* JM109/pMAL-c2x-SCO6992 before induction; 4, *E. coli* JM109/pMAL-c2x-SCO6992 after induction; 5, cell-free extract of *E. coli* JM109/pMAL-c2x-SCO6992 after induction; 6, sample after being passed through column; 7, sample after washing the column; 8, purified MBP-SCO6992 protein. (**B**) Circular dichroism (CD) spectroscopy of MBP-SCO6992 in the presence of ZnCl_2_. Scanning was performed at a range of 200 and 260 nm and a scan rate of 100 nm per minute.

To investigate its biochemical function, we tried to express SCO6992 in *E. coli* in various ways. Due to the difficulty in forming inclusion bodies of the protein alone, an MBP-SCO6992 fusion protein was constructed, by linking SCO6992 (39.5 kDa) to MBP (42.5 kDa), to obtain a soluble form in *E. coli*. The soluble MBP-SCO6992 fusion protein was successively purified by amylose affinity column chromatography. The purified protein had an approximated molecular weight of 82 kDa, as predicted (Fig. 4A).

### Analysis of the Metal Binding Ability of the MBP-SCO6992 Protein

Whether SCO6992, which has a metal binding motif, could bind metal ions was investigated using CD spectroscopy. The MBP-SCO6992 protein showed its minimum CD value (mdeg) between 210 and 222 nm. However, after reacting with Zn^2+^, the intensity of the CD band within this wavelength range decreased, suggesting that the secondary structure of the protein had changed after binding Zn^2+^ (Fig. 4B). Furthermore, no significant changes in the CD spectrum were observed that could indicate that the protein was binding any other of the metal ions tested (Fe^2+^, Mn^2+^, Mg^2+^) (data not shown). These data suggested that SCO6992 might be a zinc-binding metalloprotein.

### Analysis of the Protease Activity of the MBP-SCO6992 Protein

**Fig. 5:**
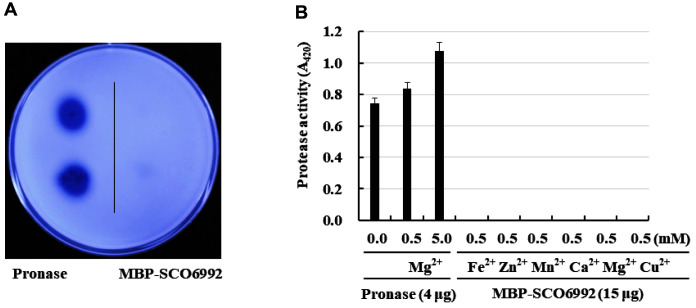
Measurement of the protease activity of MBP-SCO6992. (**A**) Skim milk hydrolysis assay. To determine its total protease activity, purified MBP-SCO6992 aliquots were dropped onto a 1% skim milk agar plate, and the formation of clear zones was observed after incubation at 37°C for 24 h. (**B**) Azocasein hydrolysis assay. The total protease activity of MBPSCO6992 toward azocasein (1%) was measured spectrophotometrically at 420 nm (A_420_) after running a reaction at 37°C for 30 min. The effect of metal ions on protease activity was also investigated by adding various metal ions to the reaction mixture. In both (**A**) and (**B**), pronase, a commercially available protease mixture, was used as the positive control. All experiments were repeated at least 3 times, and their average values were calculated.

Based on *in silico* analyses and the confirmation of its zinc-binding ability, SCO6992 was presumed to be a zinc-dependent metalloprotease. In a protease activity test (Fig. 5A), pronase, a commercially available protease mixture, showed a large hydrolysis zone in skim milk agar plate tests, whereas SCO6992 did not show any hydrolysis activity. When the protease activity was measured toward azocasein, pronase also showed a strong activity that increased by the presence of Mg^2+^. In contrast, no protease activity was shown by SCO6992 regardless of the presence or absence of metal ions, including Zn^2+^ (Fig. 5B). The above reactivity trend was the same for other substrates such as artificial substrates for trypsin and chymotrypsin (data not shown). Therefore, we determined that SCO6992 was not a metalloprotease.

### β-Glucuronidase Activity of SCO6992

**Fig. 6:**
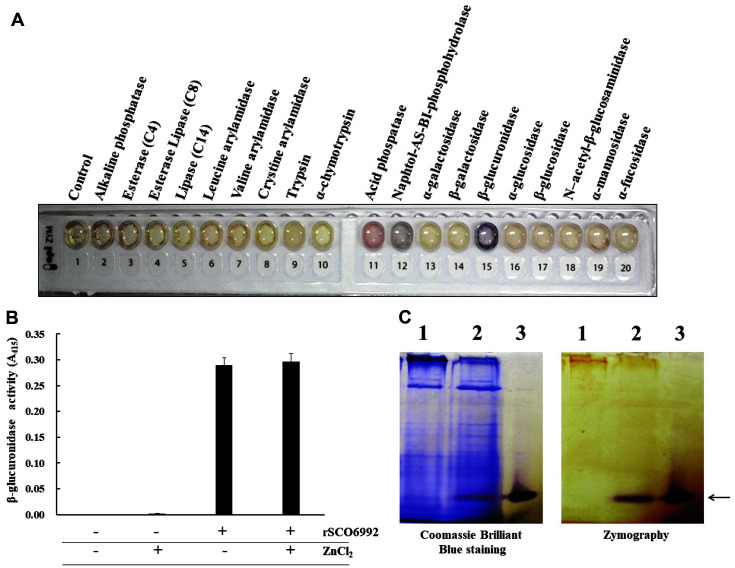
β-glucuronidase activity of MBP-SCO6992. (**A**) Detection of β-glucuronidase activity. An API ZYM kit (bioMérieux) was used to investigate the enzymatic activity of the purified protein. MBP-SCO6992 showed a strong β- glucuronidase activity and a week acid phosphatase activity. (**B**) Measurement of β-glucuronidase activity. The artificial substrate *p*-nitrophenyl-β-D-glucuronide was used to measure β-glucuronidase activity. After incubating the enzymatic reaction at 37°C for 1 h, the concentration of p-nitrophenol, a product of the hydrolysis, was measured at 415 nm (A_415_). MBPSCO6992 showed strong hydrolyzing activity against the substrate used, and showed a slight increase in activity by the addition of Zn^2+^ ions. All experiments were repeated at least 3 times, and their average values were calculated. (**C**) Zymographic analysis for β-glucuronidase. The protein samples were electrophoresed onto a 9% native polyacrylamide gel. Then, the gel was immersed in 0.2 M sodium acetate buffer (pH 5.2, 100 ml) containing 40 mg of naphthol AS-BI-β-D-glucuronide (an artificial substrate) and 40 mg of Fast Garnet. The color change on the zymogram was recorded. Lanes: 1, cell free extract of *E. coli* JM109/pMAL-c2x after IPTG induction; 2, *E. coli* JM109/pMAL-c2x-SCO6992 after IPTG induction; 3, purified SCO6992- MBP. Both overexpressed and purified MBP-SCO6992 proteins show β-glucuronidase activity (indicated by an arrow).

In an attempt to identify any enzymatic activity of the SCO6992 protein, we used an API ZYM kit, which analyzes 19 different enzymatic activities, and is routinely used for microbial identification. As a result, a weak acid phosphatase activity and a strong β-glucuronidase activity were detected in MBP-SCO6992 (Fig. 6A). To confirm these results, we further searched for acid phosphatase activity using 2-naphthyl phosphate, an artificial substrate, but no activity was observed regardless of the presence or absence of Zn^2+^ ions (data not shown). However, when measuring β-glucuronidase activity using *p*-nitrophenyl-β-D-glucuronide as substrate, a strong activity was detected (Fig. 6B). Noteworthy, the β-glucuronidase activity of MBP-SCO6992 was slightly increased by the presence of Zn^2+^ ions, but the difference was not significant.

Moreover, a zymographic analysis on native polyacrylamide gel confirmed that the purified MBP-SCO6992 protein had β-glucuronidase activity on an artificial substrate such as AS-BI-β-D-glucuronide. Especially, the active band was not observed in the total protein samples obtained from *E. coli* JM109/pMAL-c2x, but was clearly observed in the total protein samples obtained from *E. coli* JM109/pMAL-c2x-SCO6992, implying that the β-glucuronidase activity of the transformant was caused by heterologous expression of MBP-SCO6992 (Fig. 6C). All these results suggest that SCO6992 is a protein having β-glucuronidase activity and has a great influence on the biosynthesis pathways of the secondary metabolism in *S. coelicolor*.

## Discussion

*S. coelicolor* is the best characterized strain at both the molecular and genetic levels among the species of the genus *Streptomyces*. Because it can produce two kinds of pigmented antibiotics, mutants which simultaneously block the biosynthesis of more than one antibiotic can be easily selected with the naked eye, and thus several classes of mutants have been reported in *S. coelicolor*. For instance, a simultaneous loss of the capacity of synthesizing antibiotics and of morphological differentiation was reported in the *bld*^-^ mutant [3]. However, mutants like *abs*^-^, *aba*^-^, and *afs*^-^ are pleiotropically defective in antibiotic synthesis but have an almost normal morphological development [7, 4, 23, 24].

In pleiotropically defective mutants, mutations in a diversity of genes have been identified, for example, mutations in genes encoding sigma factors [25], DNA-binding proteins [26], tRNAs [27], RNase III [28], protein kinases [10], methyltransferases [29], adenylate cyclase [30], or S-Adenosyl-L-methionine synthetase [31], implicating that both the morphological and physiological differentiation of *Streptomycetes* are affected by various factors. Moreover, some global regulatory genes can function beyond species barriers. For example, the AbsA1/AbsA2 and AfsQ1/AfsQ2 two-component protein systems are both composed of His-Asp kinases and exert their ability to stimulate antibiotic production in *S. coelicolor* as well as in *S. lividans* [14, 6, 32]. The global regulatory proteins, AfsR/AfsK, which are Ser/Thr kinases [12, 33], and PtpA phosphotyrosine protein phosphatase [34] also exert similar effects on *S. coelicolor* and *S. lividans*.

In this study, we cloned a DNA fragment that restored the ability to produce four antibiotics in *S. coelicolor* abs8752, a new *abs*^-^-type mutant [11]. Of the two putative genes in the clone, *SCO6992* but not *SCO6993* could stimulate antibiotic production in *S. coelicolor* strains. Furthermore, we observed that *SCO6992* also stimulated the production of pigmented antibiotics in *S. lividans* TK24 to the same extent as in *S. coelicolor* (data not shown). Phylogenetically, *S. lividans* is known to be closely related to *S. coelicolor*, but the biosynthetic genes for actinorhodin and undecylprodigiosin commonly found in *S. lividans* are usually silent [23]. Like many regulatory genes, such as *afsR*, *afsK*, *afsQ*, *abs*A, and *ptpA*, *SCO6992* highly induced antibiotic biosynthesis in *S. lividans*, leading us to conclude that SCO6992 played a global regulatory role in antibiotic production in both *Streptomyces* strains.

The presence of a metalloprotease domain with a putative metal binding motif led us to assume that SCO6992 might be a metalloprotease. Given that several studies have reported that the expression of some proteases have a great influence on both morphological differentiation and the secondary metabolism of *Streptomyces* strains [17, 35], our hypothesis seemed plausible. However, no metalloprotease activity was detected in the purified MBP-SCO6992 fusion protein. Instead, we concluded that SCO6992 is a β-glucuronidase through enzymatic and zymographic assays.

β-glucuronidases belong to the glycosidase family 2 of proteins, and are known to hydrolyze complex carbohydrates. In humans, β-glucuronidases hydrolyze glycosaminoglycans, releasing β-D-glucuronic acid residues from their non-reducing end. Increasing pathological evidence has indicated that β-glucuronidases in humans may be sensitive indicators for early identification of cell damage. In fact, an increase in β-glucuronidase levels has been reported in many pathological conditions, such as liver cirrhosis, inflammations, cholestatic jaundice, neoplasms, and tuberculosis [36].

Currently, functional studies on β-glucuronidases in prokaryotes seem to be very rare. The *gusA* gene (NP_416134) encoding β-glucuronidase (GUS) in *E. coli* has been widely used as a reporter gene to measure gene expression levels from specific promoters. However, studies on β-glucuronidase enzymes or D-glucuronic acid in *Streptomyces* are not available in Medline. Rather, it has been reported that *Streptomyces* does not have any GUS [37], implying that GusA homologues have not been found in this genus.

The amino acid sequences of SCO6992 (359 aa long) and GUS from *E. coli* (603 aa long) are not similar. Moreover, SCO6992 does not show any homology with any of the previously reported β-glucuronidases. Therefore, SCO6992 should be considered as a novel protein with β-glucuronidase activity. Interestingly, gusA from *E. coli* has been frequently used as a reporter gene in *S. lividans* TK24 [37, 38] and *S. coelicolor* [39], however its ability to stimulate pigment production has never been reported.

We placed a paper disc soaked in D-glucuronic acid on a plate spread with spores of *S. coelicolor* and *S. lividans*, and searched for changes in pigment production while incubating it. However, no significant difference in color or growth was detected around the disc (Fig. S1). This suggests that the effect of SCO6992 on stimulating antibiotic production is not due to D-glucuronic acid, but to other still unknown substrate modified by the enzyme.

Current knowledge cannot explain how the β-glucuronidase activity of the SCO6992 protein stimulates secondary metabolic pathways. Therefore, studies searching for the intracellular substrate of the SCO6992 protein and on the metabolic pathway linking such substrate with the biosynthesis of antibiotics are needed. These efforts will provide new clues to the functional identification of SCO6992 orthologues, presently listed as hypothetical proteins.
